# Littre’s hernia in a Tunisian emergency department: a rare image

**DOI:** 10.11604/pamj.2020.37.164.10735

**Published:** 2020-10-15

**Authors:** Najla Feriani, Hassen Ben Ghezala

**Affiliations:** 1 Service Universitaire de Chirurgie Générale, Hôpital Régional de Zaghouan, Faculté de Médecine de Tunis, Tunis, Tunisie,; 2 Service Universitaire des Urgences et de Réanimation Médicale, Hôpital Régional de Zaghouan, Faculté de Médecine de Tunis, Tunis, Tunisie

**Keywords:** Meckel diverticulum, complication, Littre hernia

## Image in medicine

Meckel diverticulum is known as the most common congenital anomaly of the gastrointestinal tract. It can also be part of the contents of Littre's hernia which was described for the first time in 1700 by Alexis Littre. We report in this work a very rare case of Littre hernia which presented with an irreducible mass in the left inguinal site, diagnosed preoperatively as incarcerated inguinal hernia. A 33 years old male, without any past medical history attended the emergency department of our hospital for acute abdominal pain in relation with an incarcerated inguinal hernia. He had fever, nausea and vomiting. The patient had no history of previous abdominal surgeries. Abdominal examination revealed a visible, soft and painful mass in the right groin. It was non-pulsatile and not reducible. A diagnosis of an incarcerated inguinal hernia was made in the emergency room and the patient was admitted immediately in the surgery department. Laboratory tests were normal. No imaging examinations were required. A large indirect hernia sac was dissected revealing a Meckel's diverticulum approximately 12 cm in length. A small amount of necrotic adipose tissue and purulent material was present. A simple diverticulectomy was performed and a Lichtenstein tension-free mesh repair was used to close the inguinal defect. The patient recovered without complication and was discharged from hospital after three days of management. He was seen in follow up ten days later and remained well.

**Figure 1:**
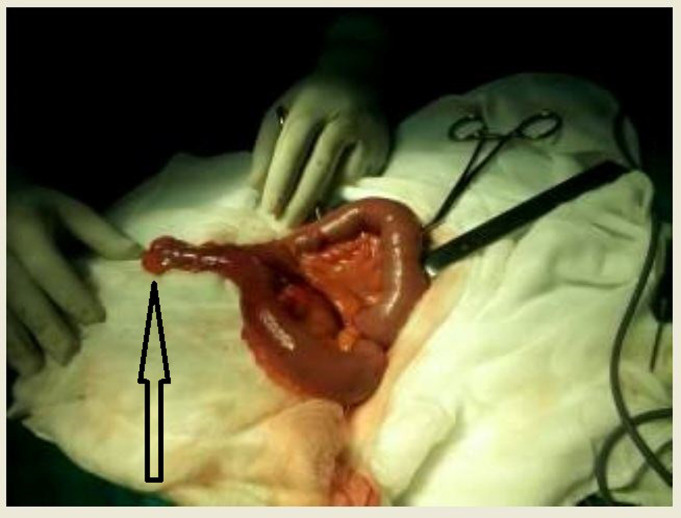
peri operative image showing a large hernia sac containing at dissection a Meckel’s diverticulum; a small amount of necrotic adipose tissue was present

